# Higher blood urea nitrogen level is independently linked with the presence and severity of neonatal sepsis

**DOI:** 10.1080/07853890.2021.2004317

**Published:** 2021-11-16

**Authors:** Xiaojuan Li, Tiewei Li, Jingjing Wang, Geng Dong, Min Zhang, Zhe Xu, Yidi Hu, Bo Xie, Junmei Yang, Yuewu Wang

**Affiliations:** a Zhengzhou Key Laboratory of Children’s Infection and Immunity, Children’s Hospital Affiliated to Zhengzhou University, Henan Children’s Hospital, Zhengzhou Children’s Hospital, Zhengzhou, China; b Department of Neonatology, Ordos Central Hospital, Ordos, China; c The Engineering Research Center for New Drug Screening, Inner Mongolia Medical University, Hohhot, China

**Keywords:** Blood urea nitrogen, neonatal sepsis, risk factor

## Abstract

**Background:**

Previous studies have demonstrated that blood urea nitrogen (BUN) is strongly associated with sepsis. However, no data are currently available regarding the association of BUN levels and neonatal sepsis. Thus, this study aimed to investigate the role of BUN in predicting the presence and severity of neonatal sepsis.

**Methods:**

In this study, we enrolled 925 neonates. Among them, 737 neonates were diagnosed with sepsis, including 426 neonates with severe sepsis. Neonates with hyperbilirubinemia (*n* = 188) served as controls. We collected complete clinical and laboratory data were collected. Multivariate logistic regression analysis was performed to identify the potential independent risk factor for neonatal sepsis. Receiver operating characteristic (ROC) curve analysis was used to evaluate the prediction accuracy of BUN in predicting neonatal sepsis. All statistical analyses were performed using the statistical package SPSS 24.0.

**Results:**

Neonates with sepsis and severe sepsis had a higher level of BUN. The prevalence of neonates with severe sepsis was dramatically increased according to BUN tertiles. Correlation analysis showed that BUN levels were positively correlated with the levels of infection marker procalcitonin (PCT) and high-sensitivity C-reactive protein (hsCRP). Multiple logistic regression analysis showed that BUN was an independent risk factor for the presence and severity of neonatal sepsis. ROC curve analysis showed that BUN had a well discriminatory power in predicting sepsis (area under curve (AUC) = 0.69, 95% CI, 0.66–0.74, *p* < .001) and severe sepsis (AUC = 0.72, 95% CI, 0.67–0.78, *p* < .001).

**Conclusion:**

Higher BUN level is independently linked with the presence and severity of neonatal sepsis.

## Introduction

Neonatal sepsis is the most common cause of morbidity and mortality in the neonatal population [1]. Early diagnosis of neonatal sepsis is helpful to give diagnostic-specific early intervention at an early stage thus avoiding unnecessary antibiotics. However, there are still some challenges in the diagnosis of neonatal sepsis, such as a long waiting time for blood culture results and non-specific clinical presentations, such as respiratory distress, pneumonia, and temperature instability [2–4]. The circulating blood levels biomarkers that may be useful in the early diagnosis of neonatal sepsis have been studied, such as procalcitonin (PCT) and high-sensitivity C-reactive protein (hsCRP) that may be useful in the early diagnosis of neonatal sepsis have been studied previously [5]. In addition, the results of our previous study showed that neutrophil–lymphocyte ratio (NLR) was an independent predictor of the presence of neonatal sepsis [6]. However, most recent studies focus mainly on the biomarkers of infection and inflammation and pay little attention to other potential biomarkers.

Blood urea nitrogen (BUN) is a waste product produced in the liver that travels through the blood to the kidneys, which then filters it out of the blood. Results of previous studies have shown that sepsis significantly decreases the renal blood flow and renal function, which can further increase the BUN levels [7–9]. Li et al. [10] and Waltz et al. [11] showed that sepsis induced kidney dysfunction and increased BUN levels in a septic mouse model. Njim et al. [12] reported that BUN was a predictor for the development of sepsis during severe malaria in adult. In addition, compared with the markers of infection and inflammation, BUN is a convenient and low-cost indicator that can reflect sepsis-induced renal injury. To data, however, the studies investigating the relationship between BUN and sepsis have been performed mostly in animals and in adult patients, and there are few published data on the relationship between the levels of BUN and neonatal sepsis. Thus, this study aimed to investigate the relationship between BUN levels and sepsis in a relatively large neonatal population.

## Materials and methods

### Study population

From January 2016 to December 2019, we retrospectively included 737 consecutive neonates diagnosed with sepsis at Henan Children’s Hospital (Children’s Hospital Affiliated to Zhengzhou University, Zhengzhou, China). In addition, we also enrolled 188 neonates who had hyperbilirubinemia as controls. The criteria for inclusion in the neonatal sepsis group were as follows: (1) neonates diagnosed with sepsis and (2) aged 1–28 d. The criteria for inclusion of neonates with hyperbilirubinemia in the control group were as follows: (1) no clinical indications of infection, such as bronchitis and pneumonia and (2) total WBC count <10 × 10^9^ cells/L and >4 × 10^9^ cells/L, and hsCRP <5 mg/L because of the concern of other infections. All neonates with the following conditions were excluded from this study: (1) the availability of a complete medical record and BUN measurements and (2) subjects with congenital diseases of the kidney, cancer, hematological system diseases and major congenital malformations. The study protocol complied with the Declaration of Helsinki and was approved by the hospital ethics review board. All procedures included in this study were undertaken as part of routine clinical practice, and the data which could identify subjects were removed. We confirmed that all the data was anonymized and maintained with confidentiality; there- fore, the requirement for informed consent has been waived because of the retrospective nature of the current study.

### Clinical evaluation and definition

According to the published International Paediatric Sepsis Consensus, neonatal sepsis is defined as suspected or proven infection accompanied with 2 or more systemic inflammatory response syndromes (SIRS) [13]. Severe sepsis was defined as sepsis in addition to one of the following conditions: cardiovascular organ dysfunction, acute respiratory distress syndrome, and dysfunction of two or more other organs [13]. The control group included neonates with hyperbilirubinemia without infection. In addition, the severity of neonatal sepsis was assessed by using the neonatal sequential organ failure assessment (nSOFA) score, which could be used as an operational definition of organ dysfunction in neonates and was associated with the risk of mortality independent of sex, pathogen, specific centre, or extreme prematurity [14,15].

### Biochemistry

Venous blood samples were collected on admission to the hospital and transported from neonatology department to laboratory. Serum BUN levels were measured using the urease glutamate dehydrogenase method (BUN kit, Maccura Biotechnology, Chengdu, China) using an automatic biochemical analyzer (AU5800 Clinical Chemistry Analyzers, Beckman Coulter, California). The levels of albumin (ALB), alanine aminotransferase (ALT), aspartate aminotransferase (AST), BUN, creatinine (CREA), total bilirubin (TBIL), total protein (TP) and uric acid (UA) were measured using an automatic biochemistry analyzer (AU5800 Clinical Chemistry Analyzers, Beckman Coulter, California) and a conventional clinical analytical method. HsCRP was detected using a latex-enhanced immunoturbidimetric assay (Ultrasensitive CRP kit, Upper Bio-Tech Shanghai, China) on an UPPER analyzer (Upper Bio-Tech, Shanghai, China). PCT levels were measured using an electrochemiluminescence-cence assay (Elecsys^®^ BRAHMS PCT kit, Roche Diagnostic, Rotkreuz, Switzerland) on a Cobas^®^ 8000 modular analyzer (Roche Diagnostic, Rotkreuz, Switzerland). HsCRP level <0.8 mg/L or PCT level >100 ng/ml or <0.02 ng/ml were considered as 0.7 mg/L, 101 ng/ml and 0.01 ng/ml, respectively.

### Statistical analysis

Continuous variables were expressed as the mean ± standard deviation (SD) or medians (interquartile range) and were analyzed using independent t-tests, one-way analysis of variance (ANOVA), or Mann–Whitney U test, as appropriate. Categorical variables were expressed as percentages (n, %) and were assessed by Chi-square or Fisher exact tests. Correlation between 2 continuous variables was examined using Pearson or Spearman correlation test. Univariate and multivariate logistic regression analysis was performed to determine the association of BUN levels with the presence and severity of neonatal sepsis. All statistical analyses were performed using SPSS 22.0 (SPSS Inc., Chicago, Illinois, USA). Prediction accuracy was evaluated using the area under the receiver operating characteristic (ROC) curves. The optimal diagnostic cut-off point was determined according to Youden’s index (sensitivity + specificity − 1). A two-sided *P* value of less than 0.05 was considered statistically significant. Figures were created using GraphPad Prism 8 (GraphPad Software Inc., San Diego, CA, USA).

## Results

### Participant characteristics

**Figure 1.**
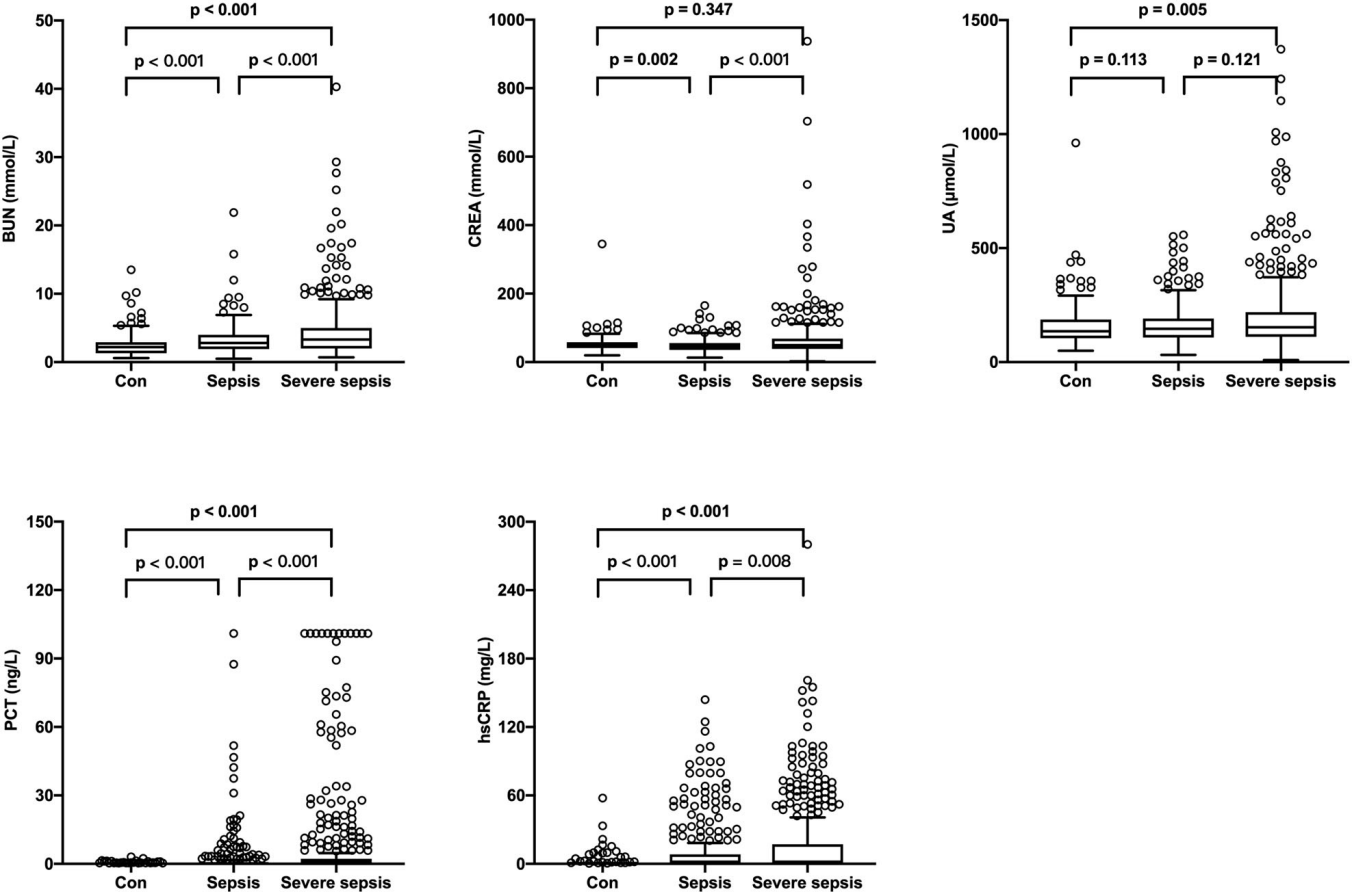
The levels of BUN, CREA, UA, PCT, and hsCRP in the control, sepsis, and severe sepsis groups. The levels of BUN, PCT, and hsCRP showed a significant gradual increase among the 3 groups.

The mean age of the 925 neonates (boys, 568 and girls, 357) was 9.0 (5.0, 16.0) days. On the basis of their diagnosis of sepsis and the severity of sepsis, the subjects were divided into 3 groups. Most of the neonates (*n* = 737, 79.7%) were diagnosed with sepsis, of those, 426 neonates were diagnosed with severe sepsis, and the remaining 188 neonates with hyperbilirubinemia were served as controls. Baseline characteristics of the neonates are shown in Table 1. Briefly, neonates with sepsis or severe sepsis had a higher body temperature, respiratory rate, and heart rate than controls (*p* < .001). Serum biochemical analysis showed that the levels of inflammatory biomarkers such as hsCRP and PCT were markedly elevated among the three groups. Additionally, our results showed that BUN levels increased gradually significantly among the three groups (Figure 1). In addition, the nSOFA was significantly higher in neonates with severe sepsis (*p* < .001).

**Table 1.** Baseline characteristics of neonates in the control, sepsis, and severe sepsis groups.

Variables	Controls (*n* = 188)	Sepsis (*n* = 311)	Severe sepsis (*n* = 426)	*p*
Age (days)	7.0 (5.0, 12.0)	11.0 (5.0, 18.0)^a^	10.0 (5.0, 16.0)^b^	<.001
Male, *n* (%)	115 (61.2%)	193 (62.1%)	260 (61.0%)	.958
Weight (kg)	3.3 ± 0.5	3.3 ± 0.6	3.1 ± 0.7b^c^	<.001
Temperature (°C)	36.9 ± 0.3	37.4 ± 0.7^a^	37.3 ± 0.8^b^	<.001
Respiratory (rate/minute)	45.4 ± 6.3	49.1 ± 10.2^a^	50.2 ± 11.5^b^	<.001
Heart rate (bpm)	141.8 ± 12.6	147.9 ± 18.7^a^	151.0 ± 20.1^b^	<.001
SBP (mm Hg)	76.9 ± 7.2	79.1 ± 5.8^a^	74.0 ± 9.2^bc^	<.001
DBP (mm Hg)	47.1 ± 7.5	47.4 ± 7.6	44.8 ± 8.2^bc^	<.001
PCT (ng/ml)	0.11 (0.09, 0.18)	0.22 (0.11, 0.76)^a^	0.42 (0.16, 2.24)^bc^	<.001
hsCRP (mg/L)	0.7 (0.7, 0.7)	0.7 (0.7, 8.3)^a^	0.7 (0.7, 17.2)^bc^	<.001
nSOFA	0.0 (0.0,0.0)	0.0 (0.0,0.0)	2.0 (0.0,3.0)^bc^	<.001
Biochemical parameters				
TBIL (μM)	306.4 (262.3, 357.7)	132.0 (47.1, 226.4)^a^	132.2 (43.9, 208.6)^b^	<.001
AST (U/L)	34.4 (28.5, 46.8)	36.4 (27.9, 49.3)	39.5 (28.0, 63.6)^bc^	<.001
ALT (U/L)	24.3 (17.6, 30.3)	28.5 (22.2, 36.1)^a^	28.4 (21.4, 39.9)^b^	<.001
TP (g/L)	57.5 ± 5.8	54.8 ± 6.2^a^	53.6 ± 7.9^b^	<.001
ALB (g/L)	34.4 ± 3.7	31.4 ± 4.6a	29.9 ± 5.0^bc^	<.001
BUN (mM)	2.2 (1.3, 2.9)	2.8 (1.9, 4.0)^a^	3.3 (2.0, 5.0)^bc^	<.001
CREA (μM)	49.6 (41.0, 57.9)	44.6 (35.8, 55.6)^a^	49.4 (38.5, 68.6)^c^	<.001
UA (μM)	135.5 (104.9, 186.5)	146.3 (108.1, 191.3)	152.6 (110.9, 219.3)^b^	.015

All Values are presented as the mean ± SD or *n* (%) or as the median (interquartile range). SBP: systolic blood pressure; DBP: diastolic blood pressure; PCT: procalcitonin; hsCRP: high sensitivity C-reactive protein; nSOFA: neonatal sequential organ failure assessment; TBIL: total bilirubin; AST: aspartate aminotransferase; ALT: alanine aminotransferase; TP: total protein; ALB: albumin; BUNA: blood urea nitrogen; CREA: creatinine; UA: uric acid. ^a^*p* <.05 for sepsis vs. control. ^b^*p* < .05 for severe sepsis vs. control. ^c^*p* < .05 for severe sepsis vs. sepsis.

### Relationship between the levels of BUN and the presence and extent of neonatal sepsis

The subjects were classified into 3 groups according to the BUN tertiles (Table 2). Neonates in the third tertile had higher levels of PCT, AST, ALT, CREA, and UA (*p* < .001). In addition, our results showed that the prevalence of neonates with severe sepsis increased significantly from 38.2% in the first tertile to 59.6% in the third tertile group (*p* < .001).

**Table 2.** Clinical and demographic characteristics according to BUN tertiles.

Variables	First tertile (<2.2) （*n* = 306）	Second tertile (2.2–3.6) （*n* = 312）	Third tertile (>3.6) （*n* = 307）	*p*
Age (days)	9.0 (6.0, 15.0)	11.0 (5.0, 17.0)	8.0 (4.0, 14.0)	.001
Male, n (%)	198 (64.7%)	190 (60.9%)	180 (58.6%)	.296
PCT (ng/ml)	0.14 (0.09, 0.27)	0.22 (0.12, 0.65)	0.66 (0.20, 4.55)	<.001
hsCRP (mg/L)	0.7 (0.7, 1.2)	0.7 (0.7, 8.2)	0.7 (0.7, 17.3)	<.001
Biochemical parameters				
TBIL (μM)	200.4 (79.9, 293.2)	156.6 (52.2, 279.7)	162.4 (55.3, 258.5)	.037
AST (U/L)	32.8 (26.6, 43.1)	37.5 (28.5, 50.3)	44.6 (30.9, 72.8)	<.001
ALT (U/L)	26.0 (20.6, 32.6)	27.1 (19.9, 36.2)	29.6 (21.5, 46.5)	<.001
TP (g/L)	53.7 ± 6.0	55.3 ± 6.5	55.4 ± 8.8^b^	.004
ALB (g/L)	30.8 ± 4.4	31.7 ± 4.4	30.5 ± 5.9	.077
CREA (μM)	45.0 (36.2, 54.3)	44.3 (35.1, 56.9)	60.2 (43.4, 84.6)	<.001
UA (μM)	115.3 (90.4, 149.6)	141.7 (112.4, 185.3)	201.6 (143.5, 319.9)	<.001
nSOFA	0.0 (0.0, 0.0)	0.0 (0.0, 0.0)	0.0 (0.0, 2.0)	<.001
Clinical data				
Con, *n* (%)	89 (29.1%)	72 (23.1%)	27 (8.8%)	<.001
Sepsis, *n* (%)	100 (32.7%)	114 (36.5%)	97 (31.6%)	.392
Severe sepsis, *n* (%)	117 (38.2%)	126 (40.4%)	183 (59.6%)	<.001

All values are presented as the mean ± SD or n (%) or as the median (interquartile range). PCT: procalcitonin; hsCRP: high sensitivity C-reactive protein; TBIL: total bilirubin; AST: aspartate aminotransferase; ALT: alanine aminotransferase; TP: total protein; ALB: albumin; BUN: blood urea nitrogen; CREA: creatinine; UA: uric acid; nSOFA: neonatal sequential organ failure assessment.

### Correlation between BUN levels and clinical parameters

Among the overall population studied, the BUN levels were negatively correlated with age (r = −0.084, *p* = .011), and positively correlated with temperature (*r* = 0.069, *p* = .036), respiratory rate (*r* = 0.089, *p* = .007), heart rate (*r* = 0.083, *p* = .012), PCT levels (*r* = 0.446, *p* < .001), hsCRP levels (*r* = .177, *p* < .001), AST levels (*r* = 0.262, *p* < .001), ALT levels (*r* = 0.172, *p* < .001), TP levels (*r* = 0.072, *p* = .028), CREA levels (*r* = 0.336, *p* < .001), UA levels (*r* = 0.486, *p* < .001), and nSOFA (*r* = 0.331, *p* < .001) (Table 3). No significant correlations were identified between the BUN levels and weight, systolic blood pressure (SBP), diastolic blood pressure (DBP), and ALB levels.

**Table 3.** Correlations between BUN levels and clinical parameters.

Variables	*r*	*p*
Age (day)	–0.084	.011
Weight (kg)	–0.024	.473
Temperature (°C)	0.069	.036
Respiratory (rate/minute)	0.089	.007
Heart rate (bpm)	0.083	.012
SBP (mm Hg)	–0.055	.096
DBP (mm Hg)	–0.037	.255
PCT (ng/ml)	0.446	<.001
hsCRP (mg/L)	0.177	<.001
AST (U/L)	0.262	<.001
ALT (U/L)	0.172	<.001
TP (g/L)	0.072	.028
ALB (g/L)	0.036	.272
CREA (μM)	0.336	<.001
UA (μM)	0.486	<.001
nSOFA	0.331	<.001

SBP: systolic blood pressure; DBP: diastolic blood pressure; PCT: procalcitonin; hsCRP: high sensitivity C-reactive protein; TBIL: total bilirubin; AST: aspartate aminotransferase; ALT: alanine aminotransferase; TP: total protein; ALB: albumin; BUNA: blood urea nitrogen; CREA: creatinine; UA: uric acid; nSOFA: neonatal sequential organ failure assessment.

### Predictive value of BUN levels for the presence and severity of neonatal sepsis

We performed univariate and multivariable binary logistic regression analysis was performed to identify the potential predictors of the presence and severity of neonatal sepsis. Variables, including age, temperature, heart rate, respiratory rate, PCT, hsCRP, TBIL, AST, ALT, TP, ALB and UA, with *p* < .05 in univariate analysis were included in the multivariate analysis. As shown in Table 4, BUN was an independent predictor of the presence of neonatal sepsis (odds ratio [OR] = 1.416, 95% confidence interval [CI] 1.207–1.662, *p* < .001) and severe sepsis (OR = 1.179, 95% CI 1.083–1.283, *p* < .001) (Table 4). Additionally, BUN tertiles were also independently associated with an increased prevalence and severity of neonatal sepsis.

**Table 4.** Regression analysis to assess the presence of neonatal sepsis and severe sepsis according to BUN levels in all neonates.

Variables	Univariate		Multivariate	
	OR (95% CI)	*p*	OR (95% CI)	*p*
Presence of sepsis				
BUN	1.364 (1.224 − 1.520)	<.001	1.416 (1.207 − 1.662)	<.001
BUN tertiles				
Tertile 1	1		1	
Tertile 2	1.367 (0.953 − 1.961)	.089	1.151 (0.696 − 1.904)	.583
Tertile 3	4.253 (2.670 − 6.776)	<.001	4.884 (2.438 − 9.786)	<.001
Presence of severe sepsis				
BUN	1.213 (1.141 − 1.289)	<.001	1.179 (1.083 − 1.283)	<.001
BUN tertiles				
Tertile 1	1		1	
Tertile 2	1.094 (0.792 − 1.511)	.584	0.938 (0.657 − 1.339)	.725
Tertile 3	2.384 (1.724 − 3.297)	<.001	1.592 (1.061 − 2.389)	.025

Adjusted for age, temperature, heart rate, respiratory rate, PCT, hsCRP, TBIL, AST, ALT, TP, ALB and UA.

BUN: blood urea nitrogen; PCT: procalcitonin; hsCRP: high sensitivity C-reactive protein; TBIL: total bilirubin; AST: aspartate aminotransferase; ALT: alanine aminotransferase; TP: total protein; ALB: albumin; UA: uric acid.

### Diagnostic performance of the BUN

**Figure 2.**
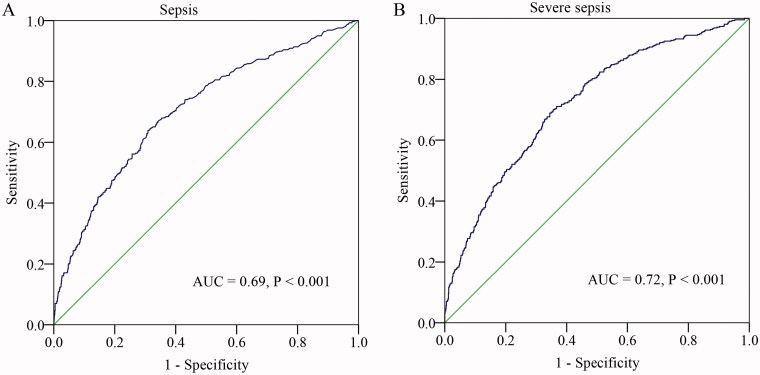
ROC curve of CPR in predicting sepsis and severe sepsis in neonates.

We performed a ROC curve analysis to evaluate the predictive value of BUN for sepsis. As shown in Figure 2, the area under the ROC curves (AUC) showed that BUN (AUC = 0.69, 95% CI, 0.66–0.74, *p* < .001) had a well predictive value for neonatal sepsis. The optimal diagnostic cut-off point was 2.6 mM, with 59% sensitivity and 86% specificity. Additionally, we examined the value of BUN in predicting severe sepsis. The AUC for the BUN in predicting severe sepsis was 0.72 (95% CI, 0.67–0.78, *p* < .001). The optimal diagnostic cut-off point was 2.8 mM (sensitivity of 65% and specificity of 80%).

## Discussion

Sepsis is a systemic inflammatory response syndrome caused by infection, and biomarkers of infection and inflammation, such as hsCRP, PCT and neutrophil extracellular traps (an emerging inflammatory marker of sepsis), play an important role in predicting the presence of neonatal sepsis [5,16,17]. In addition to the dysregulated immune response, sepsis can further lead to secondary multiple organ dysfunction syndromes [18]. Previous studies showed that sepsis induced renal blood flow and renal dysfunction, and acute kidney injury frequently occurs in patients with sepsis, which can further contribute to the burden of sepsis [11,19,20].

BUN is a waste product produced in the liver that travels through the blood to the kidneys, which then filters it out of the blood. Currently, BUN is commonly used as a marker for determining kidney function. Animal studies showed that BUN levels increased significantly in mouse models of caecal ligation and puncture (CLP)-, LPS- or Escherichia coli-induced sepsis [21–25]. Clinical studies showed that adult patients with sepsis had a higher level of basal rate of BUN production [26–28]. To date, however, the studies on the relationship between BUN levels and sepsis in neonates has been performed thus far. Compared with adults, children, particularly newborns, the kidney is still maturing, especially newborns. Studies have reported that the developing human and animal kidneys have lower renal blood flow (RBF) and higher renal vascular resistance than the mature kidney [9,29], which the kidneys may be more vulnerable to sepsis.

In this study, we firstly evaluated the relationship between the BUN levels and sepsis in neonates. Consistent with the results reported previously in adults [26–28], the results of our study showed that the BUN levels were significantly higher in neonates with sepsis and severe sepsis. In addition, our results showed that BUN levels were significantly positively correlated with the levels of the infection marker PCT and the levels of inflammation marker hsCRP, while the method for detecting BUN levels is simple and inexpensive compared with PCT and hsCRP. In addition, BUN also had a positive correlation with nSOFA. Further analysis showed that BUN was an independent risk factor of the presence and severity of neonatal sepsis. Further, we found that BUN was still an independent predictor for the presence and severity of neonatal sepsis when PCT and hsCRP levels were added to the multivariate regression model. In addition, our data also showed that BUN has a high predictive value in predicting sepsis.

Our study has several limitations in the present study. First, this is a retrospective study performed in a single centre. Second, we did not have the related data related to parameters that may affect the BUN (such as urine output, fluid chart, daily intake of protein and type of feeding, as that information was not routinely recorded in the medical records. Third, we did not track the future clinical outcomes in the present study. Further, we excluded several baseline parameters without statistical significance under univariate analysis to avoid overfitting for multivariate analysis.

## Conclusion

Higher BUN level is positively and independently associated with the presence and severity of neonatal sepsis. These findings imply that serum BUN levels can be used to predict the risk of the presence and severity of neonatal sepsis.

## Data Availability

The authors confirm that the data supporting the findings of this study are available within the article.

## References

[1] Shane AL, Sánchez PJ, Stoll BJ. Neonatal sepsis. Lancet. 2017;390(10104):1770–1780. 28434651 10.1016/S0140-6736(17)31002-4

[2] Scheer CS, Fuchs C, Gründling M, et al. Impact of antibiotic administration on blood culture positivity at the beginning of sepsis: a prospective clinical cohort study. Clin Microbiol Infect. 2019;25(3):326–331. 29879482 10.1016/j.cmi.2018.05.016

[3] Lamy B, Dargère S, Arendrup MC, et al. How to optimize the use of blood cultures for the diagnosis of bloodstream infections? A state-of-the art. Front Microbiol. 2016;7:697. 27242721 10.3389/fmicb.2016.00697 PMC4863885

[4] Mukhopadhyay S, Puopolo KM. Risk assessment in neonatal early onset sepsis. Semin Perinatol. 2012;36(6):408–415. 23177799 10.1053/j.semperi.2012.06.002 PMC3782302

[5] Sharma D, Farahbakhsh N, Shastri S, et al. Biomarkers for diagnosis of neonatal sepsis: a literature review. J Maternal-Fetal Neonatal Med. 2018;31(12):1646–1659. 10.1080/14767058.2017.1322060 28427289

[6] Li T, Dong G, Zhang M, et al. Association of neutrophil-lymphocyte ratio and the presence of neonatal sepsis. J Immunol Res. 2020;2020:1–8. 10.1155/2020/7650713 PMC7728472 33344658

[7] Langenberg C, Wan L, Egi M, et al. Renal blood flow in experimental septic acute renal failure. Kidney Int. 2006;69(11):1996–2002. 16641923 10.1038/sj.ki.5000440

[8] Toth-Heyn P, Drukker A, Guignard JP. The stressed neonatal kidney: from pathophysiology to clinical management of neonatal vasomotor nephropathy. Pediatr Nephrol. 2000;14(3):227–239. 10752764 10.1007/s004670050048

[9] Seely KA, Holthoff JH, Burns ST, et al. Hemodynamic changes in the kidney in a pediatric rat model of sepsis-induced acute kidney injury. Am J Physiol Renal Physiol. 2011;301(1):F209–217. 21511700 10.1152/ajprenal.00687.2010 PMC3129882

[10] Li JL, Li G, Jing XZ, et al. Assessment of clinical sepsis-associated biomarkers in a septic mouse model. J Int Med Res. 2018;46(6):2410–2422. 29644918 10.1177/0300060518764717 PMC6023044

[11] Waltz P, Carchman E, Gomez H, et al. Sepsis results in an altered renal metabolic and osmolyte profile. J Surg Res. 2016;202(1):8–12. 27083942 10.1016/j.jss.2015.12.011

[12] Njim T, Dondorp A, Mukaka M, et al. Identifying risk factors for the development of sepsis during adult severe malaria. Malar J. 2018;17(1):278. 30064433 10.1186/s12936-018-2430-2 PMC6066934

[13] Goldstein B, Giroir B, Randolph A. International pediatric sepsis consensus conference: definitions for sepsis and organ dysfunction in pediatrics. Pediatr Critical Care Med. 2005;6(1):2–8. 15636651 10.1097/01.PCC.0000149131.72248.E6

[14] Wynn JL, Polin RA. A neonatal sequential organ failure assessment score predicts mortality to late-onset sepsis in preterm very low birth weight infants. Pediatr Res. 2020;88(1):85–90. 31394566 10.1038/s41390-019-0517-2 PMC7007331

[15] Fleiss N, Coggins SA, Lewis AN, et al. Evaluation of the neonatal sequential organ failure assessment and mortality risk in preterm infants with late-onset infection. JAMA Netw Open. 2021;4(2):e2036518. 33538825 10.1001/jamanetworkopen.2020.36518 PMC7862993

[16] Li T, Zhang Z, Li X, et al. Neutrophil extracellular traps: signaling properties and disease relevance. Mediators Inflamm. 2020;2020:9254087. 32774152 10.1155/2020/9254087 PMC7407020

[17] Li T, Li X, Wei Y, et al. Predictive value of c-reactive protein-to-albumin ratio for neonatal sepsis. JIR. 2021;14:3207–3215. 10.2147/JIR.S321074 PMC8286121 34285544

[18] Lelubre C, Vincent JL. Mechanisms and treatment of organ failure in sepsis. Nat Rev Nephrol. 2018;14(7):417–427. 29691495 10.1038/s41581-018-0005-7

[19] Zarbock A, Gomez H, Kellum JA. Sepsis-induced acute kidney injury revisited: pathophysiology, prevention and future therapies. Curr Opin Crit Care. 2014;20(6):588–595. 25320909 10.1097/MCC.0000000000000153 PMC4495653

[20] Prowle JR. Sepsis-associated aki. Clin J Am Soc Nephrol. 2018;13(2):339–342. 29070523 10.2215/CJN.07310717 PMC5967431

[21] Karbian N, Abutbul A, El-Amore R, et al. Apoptotic cell therapy for cytokine storm associated with acute severe sepsis. Cell Death Dis. 2020;11(7):535. 32669536 10.1038/s41419-020-02748-8 PMC7363887

[22] Oh BM, Lee SJ, Park GL, et al. Erastin inhibits septic shock and inflammatory gene expression via suppression of the nf-kappab pathway. J Clin Med. 2019;8(12):2210. 10.3390/jcm8122210 PMC6947339 31847346

[23] Park JH, Park HJ, Lee SE, et al. Repositioning of the antipsychotic drug tfp for sepsis treatment. J Mol Med. 2019;97(5):647–658. 30848296 10.1007/s00109-019-01762-4 PMC6488556

[24] Chauhan AK, Kim J, Lee Y, et al. Isorhamnetin has potential for the treatment of *Escherichia coli*-induced sepsis. Molecules. 2019;24(21):3984. 31689976 10.3390/molecules24213984 PMC6864442

[25] Huang HN, Pan CY, Su BC, et al. Epinecidin-1 protects against methicillin resistant staphylococcus aureus infection and sepsis in pyemia pigs. Mar Drugs. 2019;17(12):693. 10.3390/md17120693 PMC6950563 31835381

[26] Shaw JH, Klein S, Wolfe RR. Assessment of alanine, urea, and glucose interrelationships in normal subjects and in patients with sepsis with stable isotopic tracers. Surgery. 1985;97(5):557–568. 3887629

[27] Pittiruti M, Siegel JH, Sganga G, et al. Determinants of urea nitrogen production in sepsis. Muscle catabolism, total parenteral nutrition, and hepatic clearance of amino acids. Arch Surg. 1989;124(3):362–372. 2493241 10.1001/archsurg.1989.01410030112019

[28] Kamar C, Ali A, Altun D, et al. Evaluation of risk factors and development of acute kidney injury in aneurysmal subarachnoid hemorrhage, head injury, and severe sepsis/septic shock patients during icu treatment. Ulus Travma Acil Cerrahi Derg. 2016;23(1):39–45. 10.5505/tjtes.2016.83451 28261769

[29] Mathur NB, Agarwal HS, Maria A. Acute renal failure in neonatal sepsis. Indian J Pediatr. 2006;73(6):499–502. 16816511 10.1007/BF02759894

